# Genetic susceptibility to Barrett’s oesophagus: Lessons from early studies

**DOI:** 10.1177/2050640615611018

**Published:** 2015-10-13

**Authors:** John M Findlay, Mark R Middleton, Ian Tomlinson

**Affiliations:** 1 Molecular and Population Genetics, Wellcome Trust Centre for Human Genetics, University of Oxford, Oxford, UK; 2 Oxford OesophagoGastric Centre, Churchill Hospital, Oxford University Hospitals NHS Foundation Trust, Oxford, UK; 3 NIHR Oxford Biomedical Research Centre, The Joint Research Office, Churchill Hospital, Oxford University Hospitals NHS Foundation Trust, Oxford, UK

**Keywords:** Barrett’s oesophagus, oesophageal adenocarcinoma, genetics, biomarkers, genetic susceptibility

## Abstract

Barrett’s oesophagus (BO) is a common condition, predisposing strongly to the development of oesophageal adenocarcinoma (OAC). Consequently, there has been considerable effort to determine the processes involved in the development of BO metaplasia, and ultimately develop markers of patients at risk. Whilst a number of robust acquired risk factors have been identified, a genetic component to these and the apparent increased susceptibility of certain individuals has long been suspected. This has been evidenced in part by linkage studies, but subsequently two recent genome-wide association studies (GWAS) have suggested mechanisms underlying the heritability of BO, as well as providing the first direct evidence at modern levels of statistical significance. This review discusses BO heritability, in addition to that of individual variants and genes reported to be associated with BO to date. Through this, we identify a number of plausible associations, although often tempered by issues of methodology, and discuss the priorities and need for future research.

## Introduction

Barrett’s, or columnar-lined, oesophagus (BO) is a common premalignant condition, conferring a considerable risk of progression to oesophageal adenocarcinoma (OAC). As both conditions become more common, the need for a better understanding of both becomes yet more pressing.^1^ At a translatable level, recent advances in high-throughput techniques have reinforced the potential for genomic biomarkers to help stratify susceptibility risk, and identify novel pathways, processes and therapeutic targets. This review summarises the results to date of genetic studies of BO, discusses the lessons learnt and suggests priorities for future research.

## Epidemiology

BO is the replacement of normal oesophageal squamous epithelium with macroscopically visible columnar epithelium, with or without demonstration of intestinal metaplasia.^2,3^ Adult prevalence of BO in the Western world is 0.5%–2.0%.^4,5^ Whilst associated with both frequency and duration of gastrooesophageal reflux disease (GORD) symptoms, many patients are asymptomatic and this relationship may be deceptive,^6^ corresponding with evidence that endoscopy-negative GORD, reflux oesophagitis and BO may in some regards be distinct processes.^7^ Additional risk factors include male gender, Caucasian ethnicity, increasing age, obesity, smoking, and a positive family history.^8^

## Barrett’s inheritance

The pathophysiology of GORD is extremely complicated, representing interactions between intrinsic oesophageal physiological factors (such as dysmotility), anatomical variants (such as hiatus hernia) and environmental risk factors.^7^ It is likely that the first two are modified by genetic variants, and in the case of BO, variants may also modulate the propensity to develop metaplasia after reflux, by mechanisms such as anti-oxidant imbalance and cytokine profiles.^9^

Although most BO cases appear sporadic,^10^ evidence for genetic predisposition is provided by studies identifying clustering of cases within families. However, whilst such ‘familial’ BO has been reported in up to 20% of first- or second-degree relatives, and an autosomal-dominant mode of inheritance with incomplete penetrance proposed, it is unlikely that this level of risk is representative of sporadic BO. How much phenotypic variation is accounted for by genetic factors is unclear. Traditionally, estimates of disease-associated variance have been made by concordance studies, ideally of twins. One such study estimated 43% of the GORD phenotype to be heritable,^11^ although no studies have been performed for BO.

However, early experiences with genome wide association studies (GWAS) have provided a novel perspective on heritability. These use the principle of linkage disequilibrium blocks (the non-random association of alleles at two or more loci) to genotype thousands of cases and controls using large single nucleotide polymorphism (SNP) arrays. GWAS suggest that most disease-associated variants exert a weak effect (typically an odds ratio (OR) <1.5 per allele)^12^ and are common (present in >5% of individuals).^13^ This underpins the ‘common disease-common variant’ (CDCV) model, which suggests that the majority of trait-associated variation occurs at highly polymorphic and conserved loci. These variants demonstrate low penetrance individually, but multiple variants and permutations combine to influence risk.^14^ However, although for some common diseases tens of loci have been identified, cumulatively these explain only a minority of heritability.^15^ The location and nature of this missing heritability is unclear; however, candidates include rare variants (by definition omitted from arrays) of moderate effect, undiscovered common variants of still weaker effect, variants not ‘tagged’ on existing arrays, epigenetics and inflated heritability estimates.

Two GWAS of BO have been performed, by the Wellcome Trust Case Control consortium (WTCCC) reporting initially in 2012,^16^ with further analysis in 2015,^17^ and the Barrett’s and Esophageal Adenocarcinoma CONsortium (BEACON) in 2013.^18^ Using linear mixed models of all SNPs in the BEACON genome-wide data, Ek et al. estimated heritability to be 35%,^19^ overlapping extensively with OAC. The WTCC GWAS found that more than 1000 variants contribute to BO phenotype by means of concordance of SNPs between unrelated individuals, and a disease score analysis.^16^ Subsequently, an estimate of 9.9% was made, considerably lower than the BEACON estimate.^17^ Future GWAS and complementary studies will undoubtedly refine these estimates, but the genetic contribution to BO susceptibility appears substantial.

## Candidate genes studies of susceptibility

At present, clinical factors are insufficient to guide selective screening for BO. However, reliable genomic biomarkers might facilitate this. Such markers include SNPs, short insertion or deletions, tandem repeat sequences and copy number variants. Prior to GWAS, the identification of such markers necessitated either a candidate gene or linkage-based approach to gene identification.

Plausible candidates are suggested by the genes involved in metaplasia, and a number of statistically significant associations (*p* < 0.05) have been reported (Table 1). However, extensive heterogeneity of association study methodology and quality limit their generalisability. Whilst approximately half of studies have used power calculations (although for effect sizes exceeding those predicted by the CDCV model), most studies have used low numbers of cases and controls, often representing opportunistic analysis of tissue not prospectively archived. Another frequent issue is multiple comparisons; studies often compare numerous genotype and haplotype permutations, but for half the associations reported it is not clear that their reported statistical significance would withstand correction for this multiple testing. Relatively few studies have used multivariate analysis to assess independence from clinical risk factors and other variants. There have also been differences as to cases-control matching, and defining controls. These reported associations must therefore be considered within this context, and with the majority assessed by only one study many may represent publication bias. Ultimately, no associations have been validated by independent studies, although one – the rs1695 A to G missense variant (Ile105Val) in glutathione s-transferase P (*GSTP*) 1 – is supported by meta-analysis.

**Table 1.** Reported germline variants associated with Barrett’s oesophagus.

Variant	Gene	Association			Ethnicity
		Variant	Wild-type	Effect variant	
Genome-wide association studies					
rs9257809 rs9936833	MHC region *FOXF1*	Su et al.^16^ 2012		A C	Caucasian
rs10419226 rs11789015 rs2687201	*CRTC1 BARX1 FOXP1*	Levine et al.^18^ 2013 Associated with either BO/OAC Associated with BO when combined with Su et al.^16^ 2012		A G T	Caucasian
rs3072 rs2771108	*GDF7 TBX5*	Palles et al.^17^ 2015		G G	Caucasian
Candidate studies					
Homeotic regulators					
rs2237091	*CDX1*		Ren et al.^20^ 2014	AA	Caucasian
rs717746	*CDX1*	Ren et al.^20^ 2014		GG	Caucasian
rs3776083	*CDX1*	Ren et al.^20^ 2014		AG	Caucasian
rs4769585	*CDX2*	Ren et al.^20^ 2014		CT	Caucasian
rs3812863	*CDX2*	Ren et al.^20^ 2014		AG	Caucasian
rs3776082	*CDX1*	Ren et al.^20^ 2014		AA	Caucasian
Inflammatory mediators					
rs1143634	*IL1B*		Izakovicoka-Holla et al.^21^ 2013	T	Caucasian
VNTR polymorphism	*IL1RN*		Izakovicoka-Holla et al.^21^ 2013	1/2	Caucasian
rs1800587/rs16944/ rs1143634/IL1RN	*IL1B/ILRN*		Izakovicoka-Holla et al.^21^ 2013	C/T/C/long	Caucasian
rs1800587/rs16944/r s1143634/IL1RN	*IL1B/ILRN*		Izakovicoka-Holla et al.^21^ 2013	T/C/C/long	Caucasian
rs909253	*TNFB*	Menke et al.^22^ 2012a		AA	Caucasian
rs917997	*IL18RAP*		Babar 2012 et al.^23^	CC	Caucasian
rs1946518	*IL18*	Babar et al.^23^ 2012		CC	Caucasian
rs11209026	*IL23R*	Gaj et al.^24^ 2008		A	Caucasians
rs3212227	*IL12B*	Moons et al.^25^ 2008		C	Caucasian
rs415958/rs16944	*IL1RA/IL1B*	Gough et al.^26^ 2005		CC/A	NS (UK)
rs1800896	*IL10*	Gough et al.^26^ 2005		CC	NS (UK)
Growth factors					
rs4444903	*EGF*	Menke et al.^22^ 2012b		GG	Caucasian
rs6214	*IGF1*		McElholm et al.^27^ 2010	AA	Caucasian
rs2229765 + BMI>30	*IGF1R*	MacDonald et al.^28^ 2009		A	Caucasian
Endotoxin handling					
rs6785049	*PXR*	van de Winkel et al.^29^ 2011		G	Caucasian
rs1695	*GSTP*	van de Winkel et al.^29^ 2009 Kala et al.^30^ 2007		G G	Caucasian Caucasian
Cell cycle regulation					
rs9344	*CCND1*	Casson et al.^31^ 2005a		AA	NS
DNA repair					
rs25487	*XRCC1*	Casson et al.^31^ 2005b		AA	NS
Cytoskeletal structure (intestinal permeability)					
rs2305764	*MyoPb*	Menke et al.^22^ 2012c		GG	Caucasian
Apoptosis, cellular adhesion, angiogenesis					
rs1799750	*MMP1*	Bradbury et al.^32^ 2009		GG	Caucasian
Antioxidant generation					
rs1800566	*NQO1*	di Martino et al.^33^ 2007		T	NS (UK)

NS: not specified; BO: Barrett’s oesophagus; OAC: oesophageal adenocarcinoma; MHC: major histocompatibility complex; VNTR: variable number of tandem repeat; BMI: body mass index; UK: unknown.

GSTP1 is a phase II enzyme involved in the handling of endotoxins. The common rs1695 A>G missense variant results in substitution of valine for isoleucine leading to reduced activity. It has been assessed by four studies,^30,34–36^ one demonstrating a statistically significant association, albeit derived from only 22 cases.^30^ In 2009, Bull et al. calculated a pooled OR for BO of 1.50 (95% confidence interval (CI) 1.16–1.95). Another assessed endotoxin-handling variant is the A>G substitution rs6785049 in the pregnane X receptor (*PXR*); however, as the *p* value was not reported, and no corrections were made for multiple comparisons, it is unclear whether this association was genuine.^29^

The caudal homeobox (*CDX*) homeotic regulators 1 and 2 have been implicated as promotors of metaplasia, and are up-regulated by exposure of oesophageal squamous epithelium to bile acids.^37^ Ten SNPs in either *CDX1* or *CDX2* were tested by Ren et al. in 2014;^20^ following correction for multiple comparisons, three *CDX1* (unadjusted) associations were reported (rs3776082 GG, rs2237091 AA and rs717767 GG variant genotypes). However, whilst associations with age, gender and hiatus hernia were demonstrated, multivariate analysis was not performed for these. Additionally, these SNPs are non-coding, and so any mechanisms of effect are not immediately apparent.

Reflux-induced inflammation is integral to BO.^9^ Numerous cytokine pathway variants have been tested, but whilst associations have been reported for variants in the IL1 cluster,^21,23,24,26^ none would appear to remain significant following adjustment for multiple comparisons, other than the wild-type rs917997 variant in *IL18RAP*,^26^ and rs3212227 A>C SNP in *IL12B*, both adjusted for clinical risk factors.^25^

Another group of candidate genes are the growth factors, heavily implicated in the metaplasia-dysplasia-adenocarcinoma sequence; similarly, just three (adjusted for clinical covariates) would appear to survive multiple comparisons: rs6214 G>A in *IGF1*,^27^ rs2229765 G>A in *IGF1R*^28^ and rs444903 in *EGF*.^22^ Other variants include rs9344 G>A in the cell cycle regulator *CCND1*, resulting in bypass by DNA-damaged cells of the G1/S phase checkpoint.^38^ An association of wild-type rs25487 G>A in the nucleotide excision repair component *XRCC1* with BO was also demonstrated by the same authors,^31^ although not supported by Ferguson et al. in a larger study.^39^ However, the involvement of cell cycle and DNA damage regulators highlights the accumulation of somatic variation and genomic instability characterising BO clonal populations.^40^

A recent model-free linkage analysis of 21 sibling pairs with either BO or OAC also searched for new candidate genes**,** by definition representing rarer but high penetrance alleles, associated with ‘familial’ rather than ‘sporadic’ BO.^41^ This identified three: *MSR1*, *ASCC1*, and *CTHRC1*. Prospective evaluation reported an association with rs41341748 (*MSR1*) with a combination of BO/OAC, although no assessment was made for BO alone. This variant is thought to disrupt the function of the class A macrophage scavenger receptor encoded by *MSR1*, with consequent effects on oxidative stress, inflammation and apoptosis. Due to the complexity involved in BO pathogenesis, it is likely that analogous variants are yet to be identified.

However, despite the apparent plausibility a priori of many of these variants, this must be tempered by the absence of validation, methodology quality and small sample sizes. We recently performed a replication analysis of 26 variants within the WTCCC GWAS discovery dataset of 1852 cases and 5172 controls; supportive evidence was apparent only for one: rs909253 in tumour necrosis factor beta (*TNFβ)* (OR 1.12 (1.03–1.21); *p* = 0.005).^17^ A number of lessons can therefore be drawn for future candidate studies: primarily the necessity for much larger populations powered for variants of more modest and plausible effect sizes, and appropriate adjustment for multiple comparisons. Additionally, robust assessment of relationships with established (and readily available) clinical risk factors will shed light on mechanisms and provide context for their use as biomarkers.

## GWAS

In contrast to candidate studies, GWAS are large, laborious and expensive, but have a number of scientific advantages. Their hypothesis-free approach allows detection of variants in unsuspected genes or regulatory loci, ancestry can be derived and used as a covariate, and pathway enrichment analysis may implicate previously unsuspected processes. As introduced above, their principle rests on linkage disequilibrium; SNPs tend to associate with each other, with each estimated to have 3 to 10 perfect proxies.^42^ This redundancy means that genotyping 500,000–1,000,000 SNPs is representative of almost all the 10 million common SNPs known, ‘tagging’ up to half the additive variation of common phenotypes.^43^ Due to the enormous number of comparisons involved, statistical significance is by convention taken at *p* < 5.00 × 10^−8^. Inevitably, variants identified may well be proxies for functional variants, although fine mapping of chromosomal regions, imputation of genotypes, annotation and functional studies can help resolve this.

To date, four intriguing variants have emerged for BO alone from the WTCCC GWAS. Initially, rs9257809 within the major histocompatibility complex (MHC; OR 1.21 (1.13–1.28); *p* = 4.09 × 10^−9^) and rs9936833, an intergenic SNP 141 kb from *FOXF1* (OR 1.14 (1.10–1.19); *p* = 2.74 × 10^−10^), were described. Subsequently, rs3072 (OR 1.14 (1.09–1.18); *p* = 1.80 × 10^−11^) and rs2701108 (OR 0.90 (0.86–0.93); *p* = 7.50 × 10^−9^) lying near *GDF7* and *TBX5* respectively were identified.^17^ Both rs9257809 and rs9936833 were subsequently associated with OAC^44^ and validated in the BEACON data.

The association with rs9257809 in the MHC at 6p21 supports the inflammatory model of BO, although attribution of precise function is made difficult by long-range regional LD. rs9936833 lies within a regulatory region affecting *FOXF1* (a forkhead family transcription factor). Whilst *FOXF1* has not previously been associated with BO, it is implicated in oesophageal embryology.^45^ The two most recently described variants appear to have similar roles, involved in inflammation (rs3072) and thoracic embryogenesis (rs2701108). rs3072 lies within an intergenic enhancer region, potentially regulating *GDF7*. *GDF7* encodes a member of the transforming growth factor-β super-family, involved in tissue development and repair and a ‘master switch’ gene family in oesophageal metaplasia.^46^ rs2701108 does not exert an obvious regulatory effect; however, imputation suggests that it ‘tags’ another variant: rs1920562. Whatever the underlying SNP, its likely target appears to be *TBX5*: a member of the highly conserved T-box transcription factors, involved in transcriptional regulation of mesodermal, thoracic and diaphragmatic development.^47^

The BEACON GWAS instead searched for SNPs associated with disease in a mixed set of BO and OAC patients. Three associations were found, each reinforcing a developmental role: rs2687201 (*FOXP1*), rs11789015 (*BARX1*) and rs10419226 (*CRTC1*).^18^ None reached the genome-wide threshold for BO alone, although rs10419226 reached 5.54 × 10^−8^. *FOXP1* encodes a transcription factor involved in oesophageal and pulmonary embryogenesis;^50^ *BARX1* similarly contributes to trachea-oesophageal embryogenesis via the Wnt pathway;^49^ *CRTC1* is a transcriptional co-activator, although its role is unclear.

We subsequently assessed these SNPs using the WTCC discovery dataset: On meta-analysis the rs2687201 association reached genome-wide significance for BO alone, that of rs11789015 improved (non-significantly), but that of rs10419226 reduced (with no association evident in our dataset). In addition, we assessed 87 SNPs with possible evidence of association (*p* < 1 × 10^−4^) in the BEACON GWAS: Whilst none demonstrated a significant association with BO one, rs3784262 (*ALDH1A2*), demonstrated an association with BO/OAC: 0.90 (0.87–0.93; *p* = 3.72 × 10^−9^).

## Pathway analysis

Another advantage of GWAS is the facilitation of pathway or gene set enrichment analysis (GSEA). This helps identify contributory biological pathways, elucidating pathogenesis, and suggesting clues as to diagnostic and therapeutic targets, and somewhat mitigating the limitations of considering genes in isolation. These include the risk that stringent correction for multiple testing may obscure genuine associations; furthermore, genes and variants function not in isolation, but within a constellation of others.

GSEA takes a priori-defined gene sets, and determines whether variants in these are randomly distributed across significance levels, or in fact deviate (as would be the case if the set contributed to the phenotype).^50^ GSEA platforms calculate an enrichment score, for gene sets and subsequently false discovery rate (FDR). We recently performed GSEA of the WTCCC GWAS data using two platforms.^17^ Whilst no pathways with FDR<0.05 were identified by both, three were associated with FDR<0.05 by one with supportive evidence by the other (FDR<0.25): the Kyoto Encyclopedia of Genes and Genomes (KEGG) type 1 diabetes mellitus, antigen processing and presentation and autoimmune thyroid disease pathways, supporting the roles of the inflammatory response in BO pathogenesis.

## Next-generation sequencing

In contrast to genotyping variants at specific base positions, DNA sequencing determines the complete sequence of nucleotides. First-generation (Sanger) sequencing was described in 1977, determining the sequence base by base.^51^ Second- (or next-) generation sequencing (NGS) techniques use massively parallel sequencing processes to sequence the exome, genome or transcriptome (messenger RNA, mRNA).^52^ Sequenced fragments are mapped, variants identified and their functional significance predicted.^53^ A handful of NGS studies have been performed in oesophageal cancer, although focussing on somatic variants. One study identified candidate driver mutations present in OAC and adjacent BO, but no dedicated BO studies have been performed.^54^ As with GWAS cost is often prohibitive, but it is likely that as techniques are refined (including single molecule sequencing^55^), they will identify new candidates and pathways for validation and functional annotation.

## Conclusion

To date, a variety of approaches, from small-scale candidate studies of solitary variants, to large-scale GWAS of many thousands of cases and controls, have advanced our understanding of the complex heritability and pathogenesis of BO. Furthermore, they have provided us with a number of plausible candidate genomic biomarkers of susceptibility. However, other than a small number of GWAS-identified variants, none is supported by sufficient quality and quantity of evidence. A concerted approach, of both complementary GWAS and rigorous candidate validations (perhaps within the robust framework afforded by multicentre clinical trials), is required to better evaluate and identify genomic biomarkers within the context of established clinical markers. From a translatable biological perspective, these associations point strongly to the role of both inflammation and development in BO. The former process in particular is amenable to intervention, as underpinning the ongoing Phase III Randomized, Study of Aspirin and Esomeprazole Chemoprevention in Barrett’s Metaplasia (AspECT) in patients with established BO. However, ultimately future studies may support the role of similar chemoprevention in selected patients prior to the development of BO.
